# Mechanical properties and durability of slag granite geopolymer cement incorporated zirconium aluminum layered double hydroxide

**DOI:** 10.1038/s41598-025-02052-5

**Published:** 2025-05-22

**Authors:** Fayza S. Hashem, Ahmed T. Abdel Salam, Dalia Monir

**Affiliations:** 1 Chemistry Department, Faculty of Science, Ain Shams University, P.O: 11566, Cairo, Egypt; 2 Faculty of Veterinary Medicine, Cairo University, Cairo, Egypt

**Keywords:** Geopolymer, Granite powder, Compressive strength, Irradiation by gamma ray, Durability, Chemistry, Energy science and technology, Engineering, Materials science, Nanoscience and technology

## Abstract

This research developed an alkali-activated geopolymer (GP) cement using powdered granite waste (GW), blast furnace slag (BFS), and Zirconium aluminum Layered double hydroxide (Zr–Al–CO_3_ LDH). The alkali-activation reactions were promoted using NaOH and Na_2_SiO_3_ (1:1) as an alkaline activator. The durability of various GP mixes was tested by examining their mechanical properties against firing up to 800 °C and exposure to high doses of gamma rays. Results indicated that incorporating up to 30% granite powder into the GP mixture resulted in faster setting times: initial setting time decreased by 20%, and final setting time decreased by 33%. This improvement is attributed to the acceleration of alkali-activation reactions and the increased stiffness of the paste, which is due to the surplus soluble silicon ions released from the granite powder. Replacing BFS with 10% GW led to an improvement in strength by approximately 4–6%. However, increasing the replacement ratio resulted in a decline in mechanical properties. Enhancing the 80% BFS and 20% granite waste (GW) mixture with 0.5–1% of Zr–Al–CO_3_ LDH significantly increased compression resistance by 37% and 25% at all stages of the alkali activation process. This enhancement in compression resistance is attributed to the nano-filling effect of Zr–Al–CO_3_ LDH and its ability to improve the bonding between the BFS and granite particles. Furthermore, BFS (blast furnace slag) reinforced with LDH (layered double hydroxide) showed no loss of strength during durability tests against gamma-ray irradiation at doses up to 1000 kGy. Additionally, it demonstrated thermal stability when fired at temperatures up to 800 °C, in contrast to the GP mix made solely from BFS. This behavior is mainly due to the combined action of granite particles, which serve as energy storage and thermal insulating materials, with the nano-filling and/or absorptivity properties of Zr–Al–CO_3_ LDH within the GP matrix which reinforces the geopolymer structure to endure the detrimental impacts of these demanding environments.

## Introduction

Portland cement (PC) is considered the most effective construction material, that can be served as a binding component in traditional concrete. PC manufacturers are encountering significant challenges, including the global rise in carbon emissions, a major contributor to climate change^1^. Alkali-activated or geopolymer cement (GP) is an inorganic polymeric material with suitable binding properties, offering potential as a substitute for traditional cement in construction mixes^2^. Geopolymers (GP) are composites with cementitious properties that do not rely on Portland cement (PC). The production of Geopolymer binders does not rely on the burning of limestone, which helps reduce CO2 emissions in the vicinity^3^. Additionally, in the production of GPs, industrial solid waste can be used as main sources of alumina and silica to minimize their potential accumulation risks in the future^4–6^. Furthermore, geopolymer (GP) composites have excellent mechanical properties and increased resistance in acidic and corrosive environments^7,8^. Moreover, these cement binders showed enhanced performance when subjected to heat or radiation^9–14^. From an artistic perspective, GP provides a cost-effective and environmentally friendly option to Portland cement (PC) for different industrial uses. Nonetheless, the chemical makeup of the GP mix is a critical factor in establishing durability and mechanical performance.

Granite is an igneous rock composed of coarse-grained quartz, feldspars, and mica in varying proportions. Granite is a massive, hard, and tough rock with a widespread construction stone throughout human history^15–17^. Granite has many applications in the ceramic industry, such as bricks and ceramic tiles, due to its thermal stability and energy storage properties^18,19^. Granite waste (GW) is a byproduct formed during granite processing, constituting about 15–20% of the total mass as waste. Granite waste powder contains a high silica content, as well as alumina, iron, and magnesium oxides^20^. These minerals are present in an active state or can be activated either thermally or chemically. Recently, crushed granite powder can serve as a filter stone in drainage projects or for building the base for roads and pavements^21,22^. Many studies showed that granite rock is one of the common shielding materials used in building construction due to its high attenuation cross-section for high-energy gamma rays and neutrons^23–25^. Granite can also be seen as a natural pozzolana, and several researchers have examined its properties as supplementary cementitious materials to OPC^26,27^.

Layered double hydroxide (LDHs) are synthetic clays that are prepared by partial substitution of divalent cations (Ca^+ 2^, Zn^+ 2^, Mg^+ 2^, Co^+ 2^, etc.) by trivalent cations (Al^+ 3^, Fe^+ 3^ etc.), and anions are intercalated into the interlayer zone to balance the net charge^28^. Numerous studies have investigated LDHs and found them to be excellent adsorbents in various fields^29,30^. Ke et al.^31^ found that Mg-Al and Ca–Al LDHs can effectively resist the penetration of chloride ions through concrete pore structures in concrete mixes. However, further exploration is needed to fully utilize the ordered structure of LDH in cementitious systems, especially Zr–Al–CO_3_ LDH. Incorporating nano-zirconia into metakaolin-based geopolymers significantly enhances mechanical properties due to their filling action^32^. Incorporating zirconium in the preparation of layered double hydroxide is expected to enhance the effectiveness of the geopolymer cement to which it is added.

This study focuses on assessing the combined impact of granite powder waste (GW) and Zr–Al–CO_3_ LDH on the production of a highly durable and high-performance geopolymer binder. A solution mixture of Na_2_SiO_3_ and NaOH (in a 1:1 ratio) was used as the activator for the GP. The physical and mechanical properties and the performance of the geopolymer mixes prepared during firing and irradiating by gamma rays are also studied.

## Experiment

### Materials characterization

**Fig. 1:**
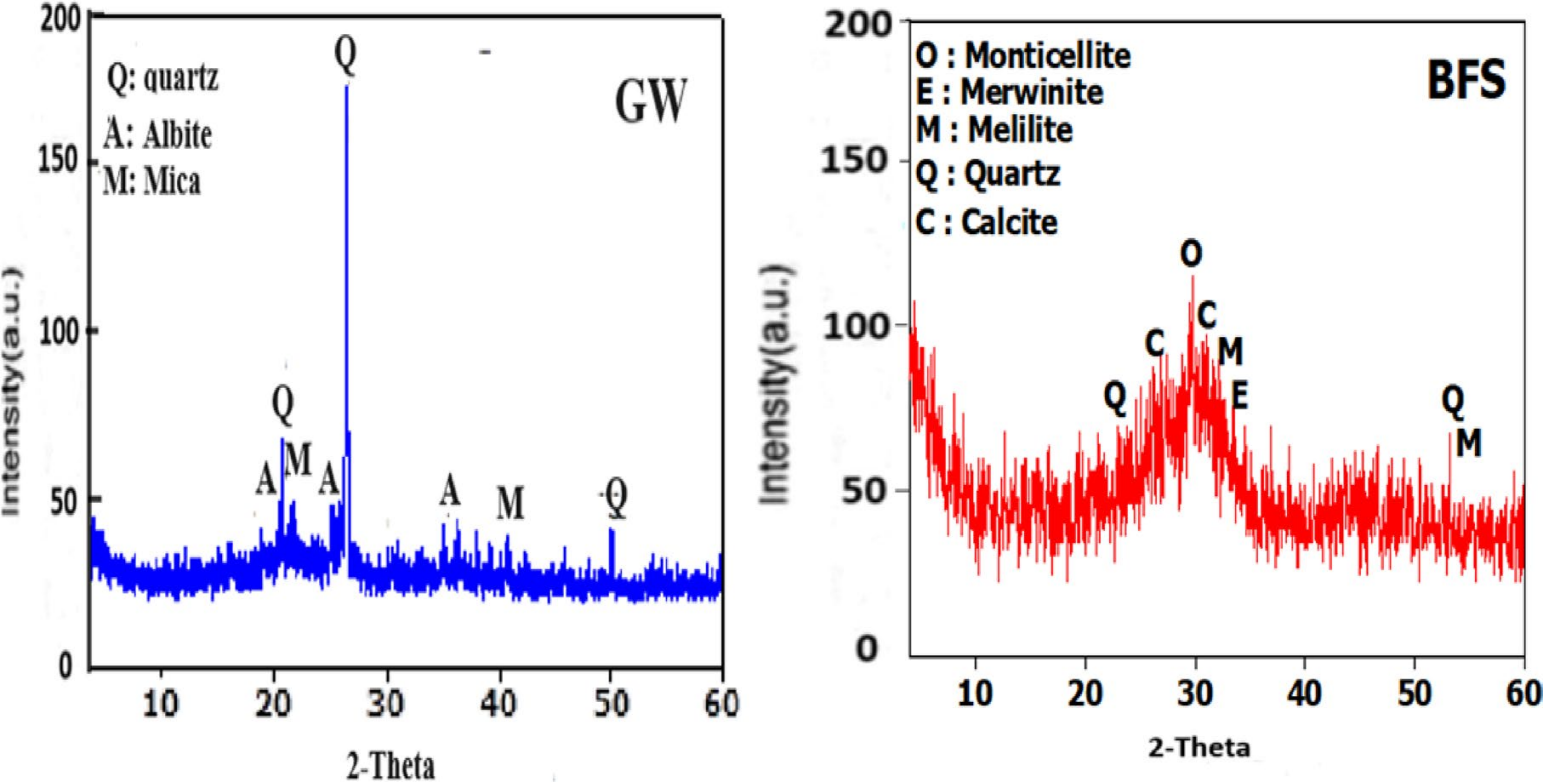
XRD of BFS and GW.

The materials used in this study were:

- Ground granulated blast-furnace slag (BFS) is sourced from an iron and steel factory located in the Helwan area of Egypt. The BFS has a Blaine-specific surface area of 2883 cm^2^/g.
- Crushed granite waste (GW) is provided from the Shuq El Thouban region. The granite powder was ground and sieved into particles sized ≤ 90 mm with a Blaine surface area of 3420 cm^2^/g. The mineralogical oxides composition (%) of BFS and GW is assessed by XRF (XRF, model PW-1400, Xios) which is represented in Table 1. Figure 1a, b. showed XRD diffractometry of BFS and GW. According to the XRF analysis of GW, SiO_2_ (68.56%) is the main oxide then Al_2_O_3_ (11.62%) and Fe_2_O_3_ (4.81%). CaO and MgO are present in low proportions. These components primarily occur as minerals such as quartz and mica in granite, as indicated in its XRD chart. These minerals contribute to granite’s thermal insulation properties. This is consistent with existing literature that highlights mica’s high thermal stability, allowing it to withstand decomposition at temperatures exceeding 1000 °C^33^. The main oxide composition of BFS is mainly CaO (38.33%), SiO_2_ (35.38%) then Al_2_O_3_ (7.11%), and MgO (5.4%). XRD diffractometry of BFS attains a broad hump between 2θ = 23–36◦, which is correlated to the amorphous and glassy nature of BFS.
- A commercial sodium silicate and sodium derived from Al-Salam Association, 6 October District, Egypt serve as alkaline activators of the prepared GP.

**Table 1:** Oxide composition of raw materials.

Oxide (%)	BFS	GW
Al_2_O_3_	7.11	11.62
CaO	35.38	2.35
SiO_2_	38.33	68.65
Fe_2_O_3_	2.96	4.81
MgO	5.40	1.24
K_2_O	0.76	0.06
Na_2_O	0.28	0.3
SO_3_	1.29	0.00
Cl^−^	0.01	0.03
Loss of Ignition (L.O.I )	9.49	10.7

### Preparation of Zr–Al–CO_3_ LDH

**Fig. 2:**
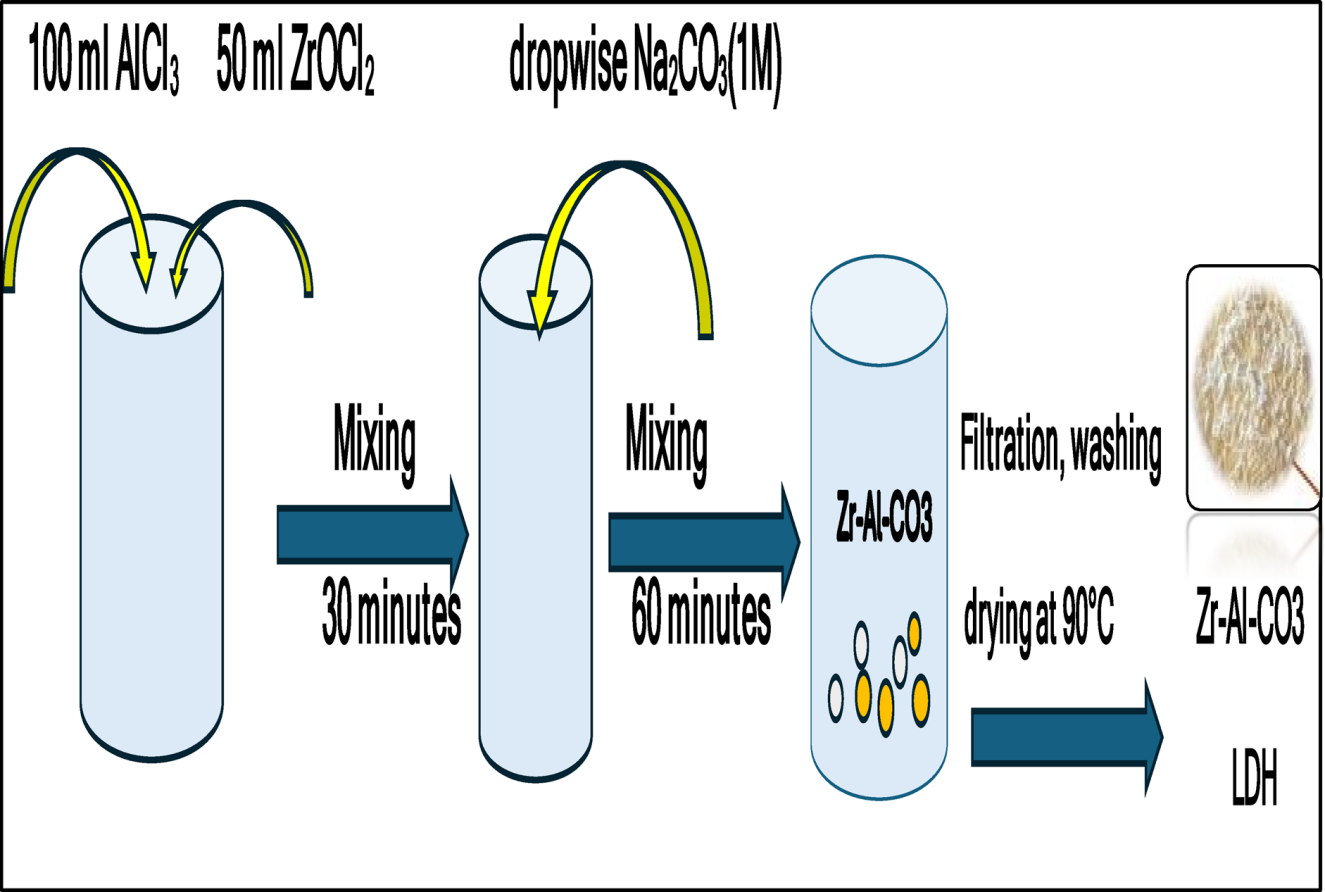
Preparation of Zr-LDH.

Zr–Al–CO_3_ LDH was prepared by coprecipitation method^34^. 50 ml of ZrOCl_2_ 8H_2_O (0.5 M) was mixed with 100 ml of AlCl_3_ (0.5 M) in a volume ratio 1:2 for half an hour.Na_2_CO_3_ (1 M) was used as a precipitating agent until a white emulsion was obtained at pH = 8. The obtained gel was washed with deionized water and dried at an oven at 90 °C overnight to complete the dryness of the LDH powders. Figure 2 shows a representation of the method of preparation of LDH. The morphology and texture characterization of the prepared LDH were examined via SEM/EDX techniques, in addition to the Brunauer-Emmett-Teller (BET) Surface Area Analysis and Barrett-Joyner-Halenda (BJH) pore size and volume analysis testing technique (BET/BJH), which will be discussed in “Characterization of Zr–Al–CO_3_ LDH” section.

### GP mix design

Different blended dry powders were produced by replacing varying proportions of ground granulated blast furnace slag (BFS) with fine granite waste (GW): 0%, 10%, 20%, and 30% by mass of BFS. A mix of 80% BFS and 20% GW was strengthened with Zr–Al–CO_3_ LDH (0.5% or 1%). A mixture solution containing equal volumes of 6 M sodium silicate and 4 M sodium hydroxide was added to the dry mix of GP. Each dry mix was then agitated for 5 h in a porcelain ball mill to ensure the uniformity of the mixture. Table 2 provides the notation and design for each dry mix. The water-to-solid ratio (W/S) in the preparation of GP pastes was 1:2 for all samples^10,11,35^. Zr–Al–CO_3_ LDH powders were added to the GP mix as suspended particles in the prepared alkaline NaOH/Na_2_SiO_3_ solution containing 0.1% naphthalene-based dispersing agent (NB)^35^ and then sonicated for 20 min (Ultrasonic- LUHS0A12–220 V/50HZ, 700 W). The suspension (NaOH/H_2_O/NB/Zn-phase) was mixed with GP powder using an automatic ELE-automatic mixer for 3 min. After mixing the dry mixes with the alkaline solution, the resulting paste was shaped into cubic samples using molds (2.5*2.5*2.5 cm) and kept at RH ≈ 98 ± 2% for 24 *hrs* to start the alkali activation reactions.

**Table 2:** Mixes notation and their composition.

Mix notation	Composition (wt,%)
M0	100% BFS
M10	90%BFS + 10%GW
M20	80%BFS + 20% GW
M30	70%BFS + 30% GW
M20-LDH1	80%BFS + 20% GW + 0.5% Zr-LDH
M20-LDH2	80%BFS + 20% GW + 1% Zr-LDH

### Testing

- The initial and final setting times for all the newly prepared geopolymer pastes were measured using the Vicat needle apparatus according to ASTM C191^36^. This apparatus consists of a frame with a movable rod with a disc on one end and a needle that can be attached to the other end.
- The compressive strength test was conducted on three cubic samples for each geopolymer mix after 3, 14, 28, and 90 days, according to ASTM C109 M16-A^37^.
- The ability of the prepared GP samples to block gamma rays was assessed on the dried 28-day sample. The examination was done by exposing the 28-days hardened samples to a gamma ray source (Co^60^ γ-cell-220, Atomic Energy Commission, India). The sample was exposed to doses of 250, 500, 750, 1000, and 1500 *kGy* at ambient temperature at a dosing rate of 1.4 *kGy/hr*^35,38^. The effect of exposing GP samples to gamma ray irradiation at varying doses on their mechanical properties was evaluated by conducting a compressive strength test, and the residual strengths (RS %) were calculated using a specific Eq. (1)^10^:
  1 $$  ~~\left( {{\text{RS}}} \right)_{{{\text{rad}}}}  = {\text{ }}\left( {{\text{CS}}} \right)_{{{\text{rad}}}} /\left( {{\text{CS}}} \right)_{0} ~~~ \times {\text{1}}00  f $$

(CS)_rad_ : is the compressive strength after irradiation by gamma ray, (CS)_0_ : is the compressive strength value before irradiation (28-day value).

-The durability of the geopolymer cement against firing was tested by exposing 28-day-old dried samples to three different firing temperatures (300 °C, 600 °C, and 800 °C) for three hours in a muffle furnace with a heating rate of 10 °C per minute^10^. These three temperatures were selected to observe the response of various GP samples and assess the durability of various cement hydrates under these fire exposure conditions^39^. After the firing test, the samples were left to gradually cooled to room temperature in closed desiccators. Subsequently, their compressive strength was tested, and the residual strength (RS)t was assessed using Eq. (2).

2 $$   {\text{(RS)t  =  ((C}}{\text{.S )t)/((C}}{\text{.S}}{\text{.)0) }} \times {\text{ 100}} $$

(C.S.)t: compressive strength after firing at heating temperature (t) .

(C.S.)0: compressive strength before firing (28-day value).

XRD (XRD, model Xpert-2000, Philips) and thermogravimetric (TGA: TA instrument, model SDT Q600) techniques were used to identify the phases formed during the hydration of various geopolymer samples. X-ray Diffraction (XRD) was conducted using a cobalt target (λ = 0.17889 nm) and a nickel filter, operating at 40 kV and 40 mA. The scanning range extended from 5° to 60° (2θ), with a scanning speed of 1 s per step and a resolution of 0.02 per step. Thermogravimetric Analysis (TGA) was performed using a TGA-TG instrument, model SDT Q600. A nitrogen environment was maintained while heating 15 mg of powdered sample (≤ 25 μm) over a temperature range of 50 °C to 1000 °C, with a heating rate of 10 °C per minute. To examine microstructural development, Scanning Electron Microscopy (SEM) was utilized, along with Energy-Dispersive X-ray Spectroscopy (EDX) for elemental analysis. The SEM device used was a Quanta 250 FEG. To prevent electron accumulation and ensure electrical conductivity, a thin layer of gold was applied to the sample prior to capturing the SEM images.

**Fig. 3:**
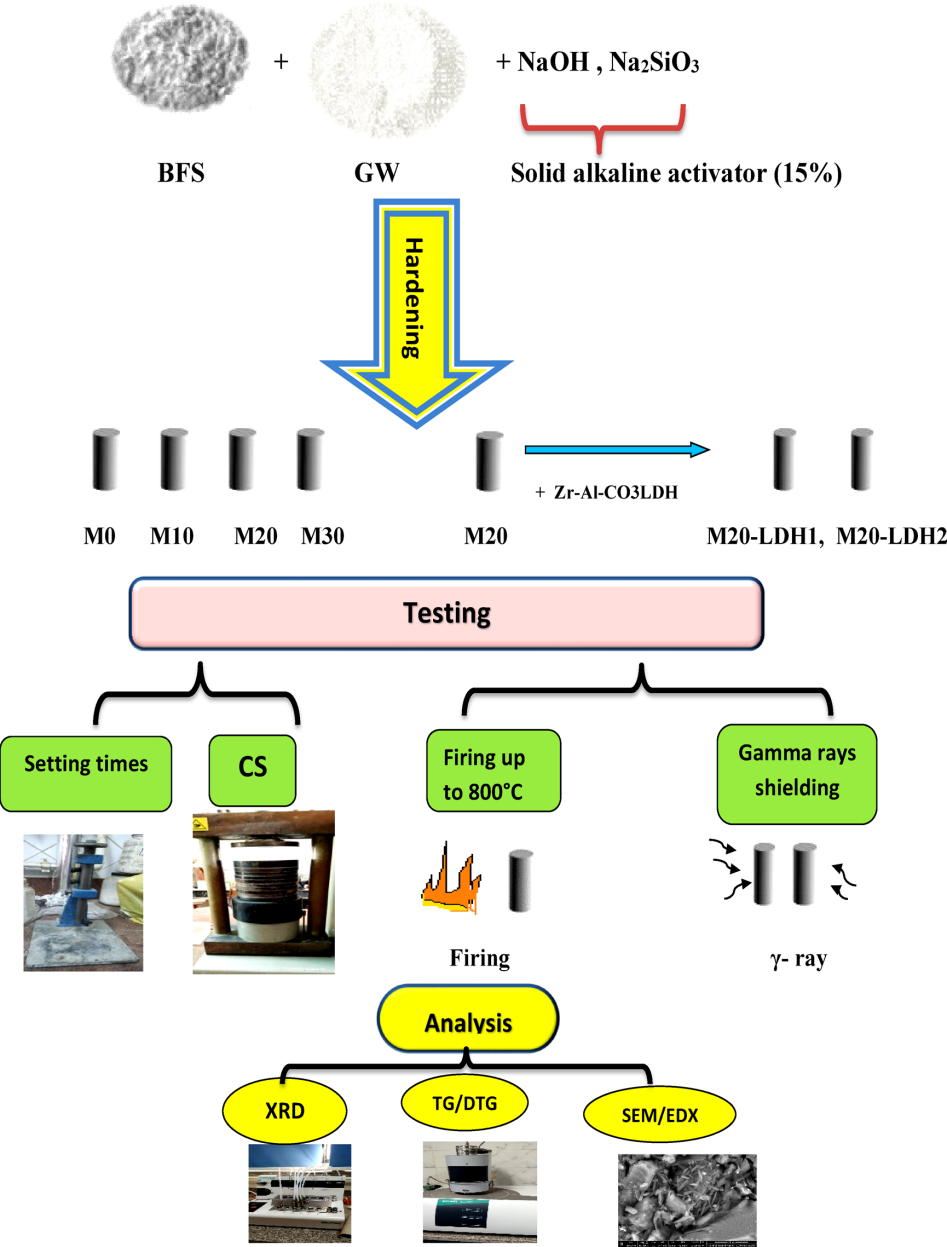
Schematic representation of the methodology.

Figure 3 provides a graphical representation of the methodology.

## Results and discussion

### Characterization of Zr–Al–CO_3_ LDH

**Fig. 4:**
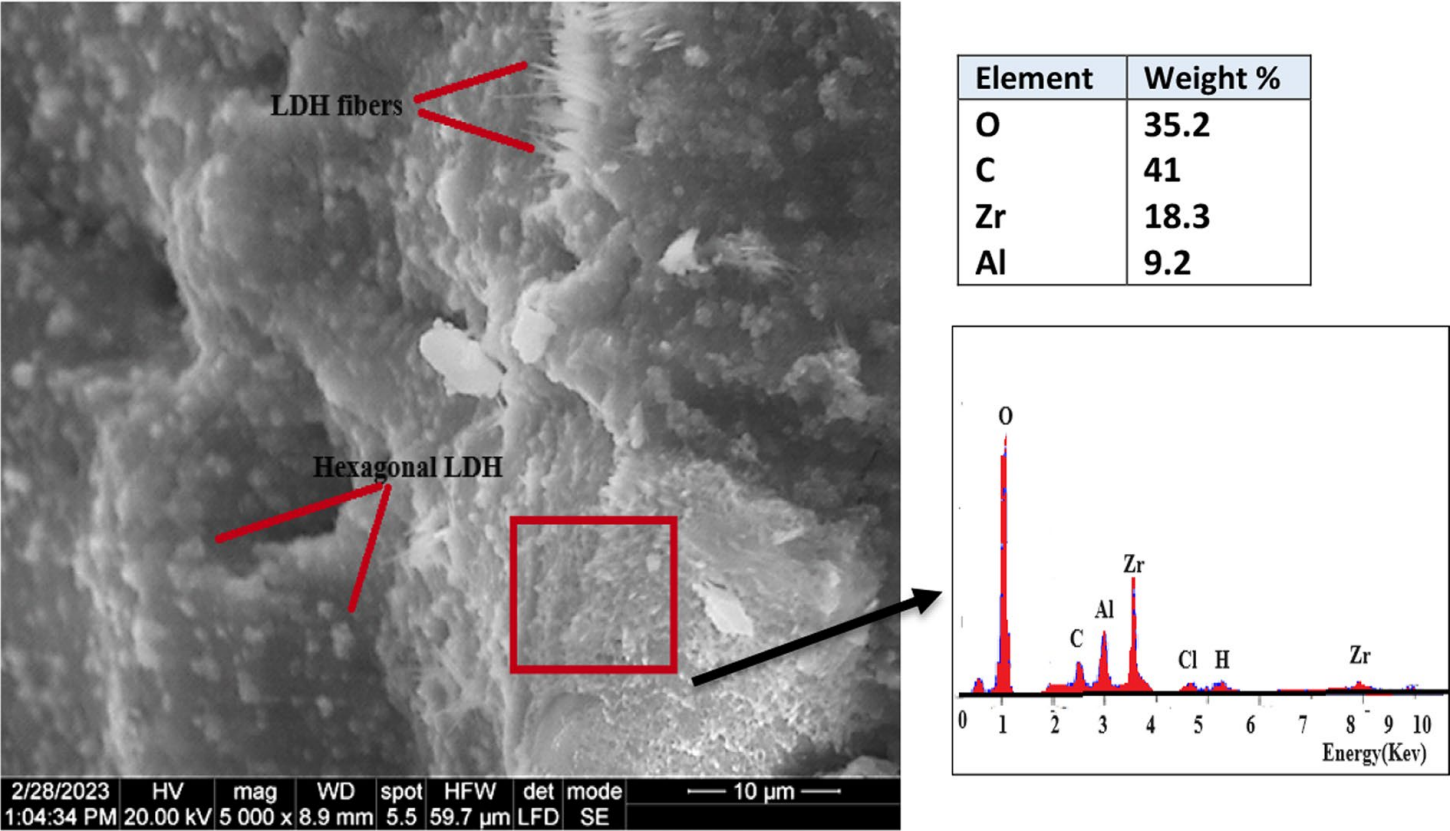
SEM/EDX image of Zr–Al–CO_3_ LDH.

The morphology and surface characteristics of the prepared Zr–Al–CO_3_ LDH were studied using SEM/EDX and N_2_-adsorption/desorption techniques. The SEM image of the Zr–Al–CO_3_LDH, shown in Fig. 4, revealed there are two crystal shapes: hexagonal crystals and fibers (which appeared as nano-rods). These two types of crystals are strongly interconnected and form a well-developed layered structure^40^. The elemental analysis (EDX spectrum) for Zr–Al–CO_3_LDH is 18.3% (Zr), 35.40% (O), 41% (C), and 9.2% (Al) which is the stoichiometric formula for Zr–Al–CO_3_ LDH^39^. (BET/BJH) models were utilized to examine the texture characterization and pore size distribution of the prepared LDH nanolayers^41^.

**Fig. 5:**
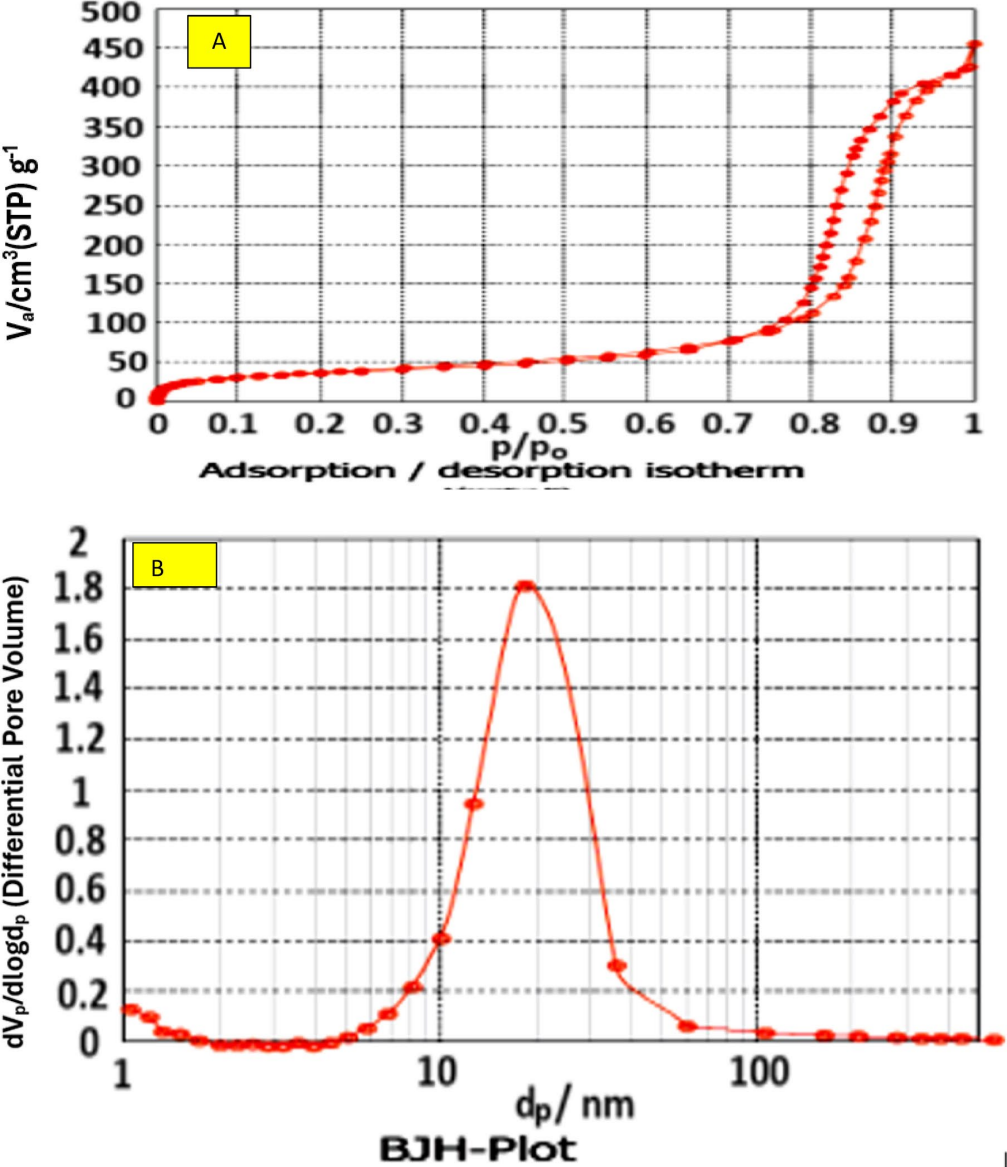
(**A**) Adsorption/desorption isotherm (**B**) BJH plot of Zr–Al–CO_3_ LDH.

Adsorption/desorption isotherm of Zr–Al–CO_3_ LDH, Fig. 5a, displays type 2 with a small H1 hysteresis loop according to IUPAC classifications which confirms the mesoporous/microporous nature of Zr–Al–CO_3_ LDH^40^. The texture parameters of Zr–Al–CO_3_ LDH were S_BET_ = 192 m^2^/g, average pore diameter = 12.77 nm, and total pore volume (Vp) = 0.677cm^3^. Figure 5b shows BJH curve for LDH which shows the maximum pore size value (dp_max_ = 28 nm). The high surface area and the tiny particle size of the prepared LDH confirm the great contribution of LDH to catalyzing the alkali-activation reactions of the created geopolymer binder.

### Setting times

**Fig. 6:**
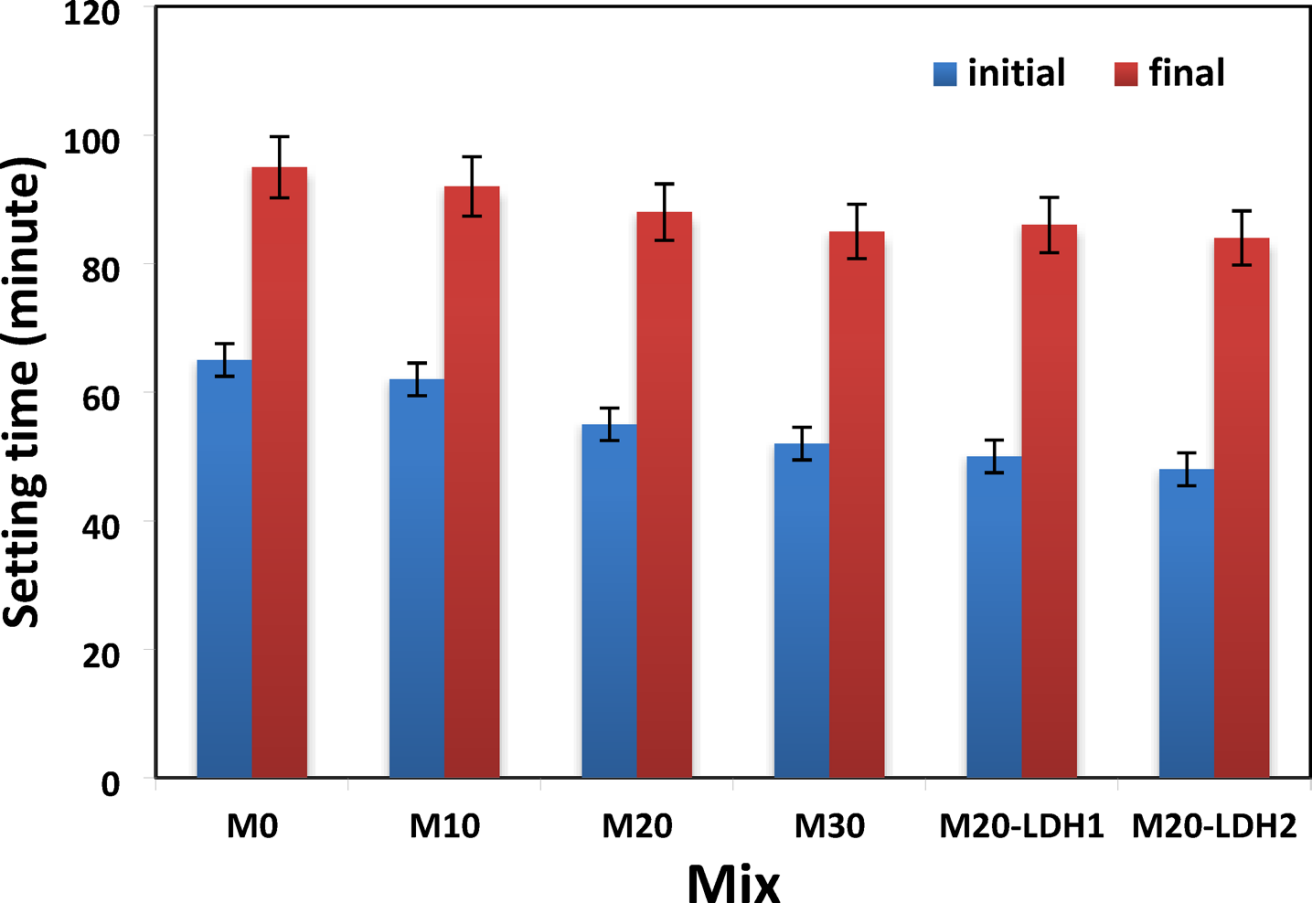
Setting times of various GP formulations.

The initial and final setting times of different GP formulations play a crucial role in determining how tested materials are handled and where they can be used. In Fig. 6, you can see that the control mix had initial and final setting times of 55 and 85 min, respectively. When ground granulated blast furnace slag (BFS) was replaced by granite powder (GW), the setting times decreased by 6 to 20% for the initial setting time and by 13 to 33% for the final setting time. The reduction in setting times is due to the high silica content in the GP formulation due to the replacement of BFS (which has a high calcium content) with GW (which has a high silica content). The elevated silica content encourages the rapid release of silica ions when the alkaline activator is introduced. The free silica ions interact with the calcium ions to initiate rapidly the formation of the GP networks. Furthermore, the high silica content in GP paste rapidly removes water from the matrix, leading to an acceleration in the setting process^10,42^. Such finding matches that of Khale and Chaudhary’s investigation, which studied the impact of silica ions on the geopolymer mechanism. They found that a high silica content facilitates the rapid release of silica ions when the alkaline activator is introduced. These ions then interact with the calcium ions to initiate the formation of the GP networks^43^.

Amelioration of GP mix by 0.5 or 1% of Zr–Al–CO_3_ layer double hydroxide results in a further reduction in the setting times to be in case mix M2-LDH1, 34 and 58 min for initial and final setting times respectively while mix M20-LDH2 recorded 31 and 55 min. This accelerated setting process is due to the acceleration impact of Al^3+^ and Zr ^2+^ metal ions released from the partial dissolution of LDH at a high pH medium. Gohi et al. clarified that at pH around 11.5, the partial dissolution of Zr-Al LDH can release Al^3+^ metal ions beside Zr^2+^ metal ions^44^. The release of Al^3+^ metal ions can contribute to forming further amounts from strength-giving-phases such as CAH and CASH^44^. Additionally, Zr–Al–CO_3_ LDH can act as active nucleation sites that catalyze the alkali-activation reaction, giving extra strength-binding phases. The impact of Zr–Al–CO_3_ layered double hydroxide (LDH) on the setting behavior of BFS/GW geopolymer is comparable to that of other nano additives, such as nano-silica and nano-clays^45^. Research has demonstrated that these materials function through similar mechanisms, primarily due to their high surface area and fine particle size, which enhance their catalytic action. This action promotes the dissolution of geopolymer oligomers and accelerates the geopolymerization process.

### Compressive strength

**Fig. 7:**
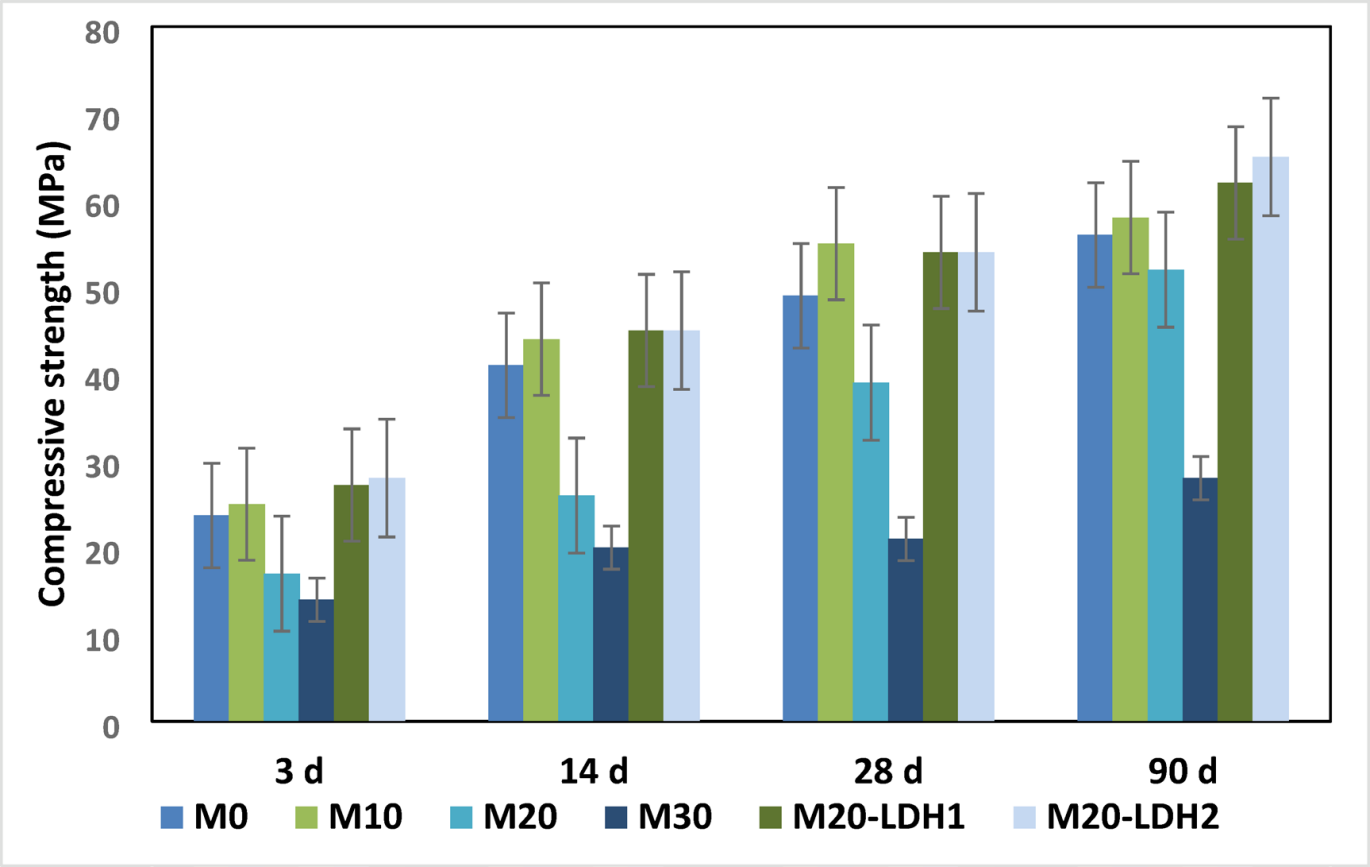
Compressive strength versus curing times for various GP mixes.

Figure 7 shows the compressive strength (measured in MPa) of various GP formulations after 3,14,28 and 90 days. The compressive strength of the control mix (100% BFS, Mix M0) increases consistently over time, with approximately 65% of the strength achieved within the first 14 days. In the initial stage of alkali activation, the alkaline mixture (blend of Na_2_SiO_3_ and NaOH) partially dissolves the BFS particles, releasing active components and facilitating the activation reactions. These reactions create strength-giving phases, such as calcium-silicate-hydrate (C-S-H) and calcium-substituted sodium-aluminosilicate-hydrate (N-C-A-S-H) gels^33,34,46^. Over time, these phases transform into a more thermodynamically stable form known as calcium-aluminate-silicate-hydrate (C-A-S-H) gel. Both calcium-silicate-hydrate and calcium-aluminate-silicate-hydrate built up the GP network and positively impacted its mechanical properties. According to the literature, forming C-A-S-H acting as micro-nucleation sites in the geopolymer gel, in which more hydrates are formed, resulting in a denser and more uniform binder^47^.

Replacing 10% of BFS with GW led to a 7 to 10% increase in strength values across all curing times. This improvement is due to the release of Si ions from the substituted granite waste powder with the shortage of Ca ions (results from BFS) in the GP system, The replacement of GW increases the Si/Al ratio while decreasing the Ca/Al ratio in the geopolymer (GP) matrix. This change promotes the formation of more N-A-S-H gel instead of C-S-H gel. According to the literature, the presence of N-A-S-H gel influences the microstructure and, consequently, the engineering properties of geopolymers. Furthermore, the more soluble silicon can interact with calcium ions from blast furnace slag (BFS), leading to the formation of greater quantities of strength-enhancing phases such as C-A-S-H and N-C-A-S-H. The N-C-A-S-H phase has a three-dimensional reticulated structure, which can create an organized two-dimensional or three-dimensional inorganic network with improved mechanical properties^48^. At 20 and 30% replacement, the compressive strength diminished by 15 and 50% for M20 and M30 respectively after 90 days. This decrease can be attributed to the dilution effect caused by blending BFS with 20% GW. This blending decreased calcium content, a key factor for promoting GP reactions, sourced from BFS which was replaced by Granite powder (has high silica content)^21^. In M20 and M30 mixes, the Ca contents were insufficient for forming considerable quantities of strength-giving phases (CSH and CASH). Shilar et al. reported that the increase in the GW content in the mix increases the compressive strength up to 10%, and beyond 20% CS declined^21^. The suitable proportion of GW in the GP mix results in more silica gel that promotes the production of denser Si–O–Si linkages during polymerization provided^49^. High GW content adversely affects the matrix structure of geopolymer composites, hindering the silica gel formation. Excess silicate delays water evaporation during polycondensation and detriment the compression resistance^50–52^.

When 80% BFS and 20% granite are mixed with 0.5% or 1% Zr–Al–CO_3_ LDH particles (as mixes M20-LDH1 and M20-LDH2), it leads to significantly improved compression resistance. The mix with a 1% addition performs better than a 0.5% addition, showing a 25% improvement in strength for M20-LDH1 and a 35% improvement for M20-LDH2 after 90 days compared to their counterparts without LDH. The improvement finding is mainly attributed to the nano-filling effect and the nucleation/catalytic properties of Zr–Al–CO_3_LDH, which result in the formation of additional hydration products that refine the microstructure. According to Vanitha et al., nano additives have been found to speed up the growth of alkali-activation gel and the filling of tiny pores^42^. It is worth noting that Zr–Al–CO_3_ LDH has a large specific surface area (192 m^2^/g) and functions as a solid binder, facilitating the connection between the BFS/granite particles^50^. Additionally, Zr–Al–CO_3_ LDH can release the CO_3_ ions which refine the GP matrix^51^. All these factors explain the increase in the strength of the M20-LDH mixes compared to their control mix.

### Durability tests

#### Gamma-ray shielding

**Fig. 8:**
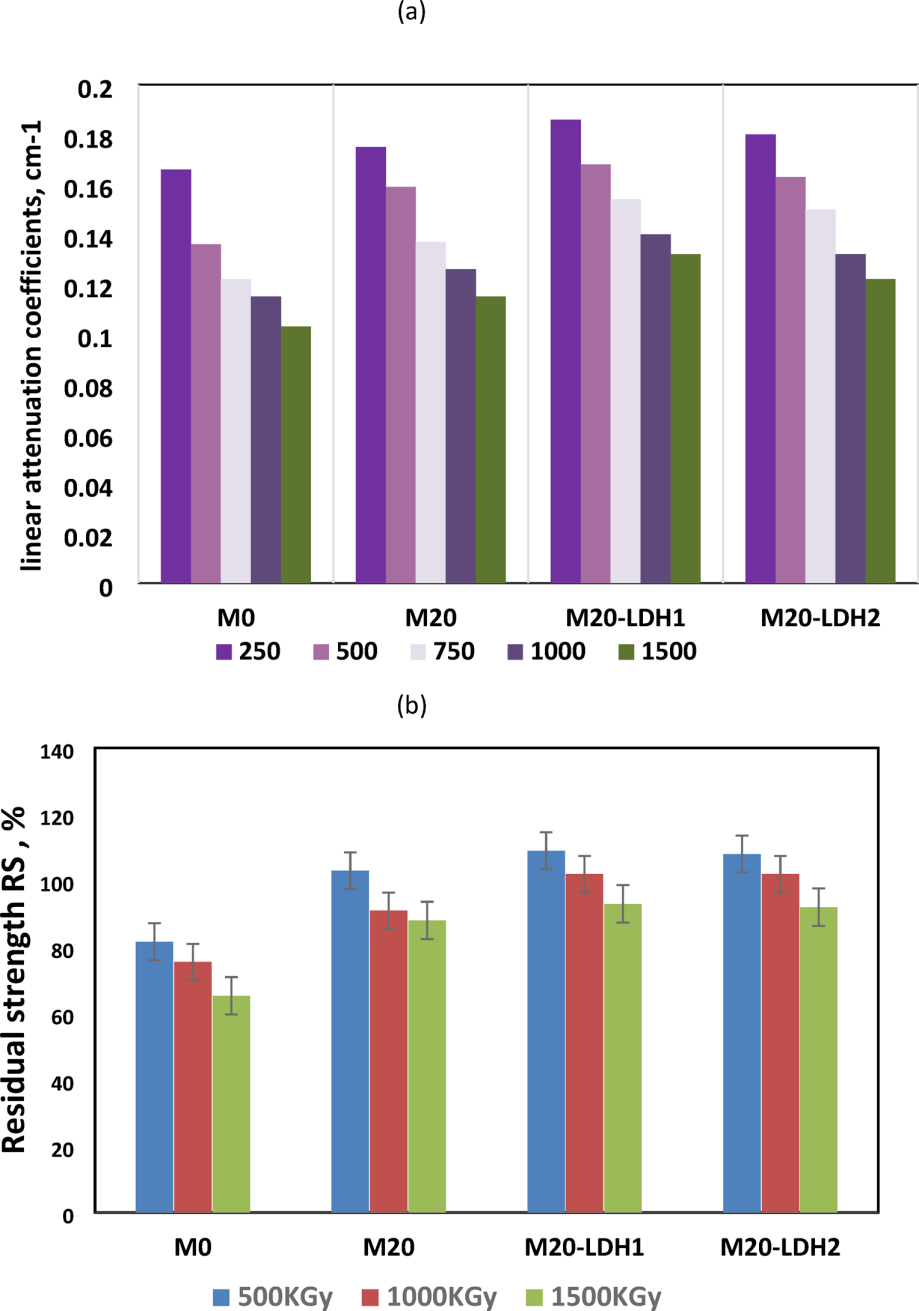
(**a**) Linear attenuation coefficient (cm^− 1^) of various GP formulations. (**b**): Residual strength (%) of various GP formulations.

The ability of construction materials to shield destructive rays, such as gamma rays, depends on their structural formulation, porosity, microstructure, their included phases, curing conditions, and the power of the applied rays^53,54^. The linear attenuation coefficient (µ, cm^− 1^) is one of the most important metrics for determining the radiation shielding capabilities of any material. The attenuation coefficient represents the fraction of an energy beam that is absorbed or scattered per unit thickness of the medium. The linear attenuation coefficients after 28 days of curing were measured to evaluate the shielding capability of M0, M20, M20-LDH1, and M20-LDH2 mixes against gamma rays. In Fig. 8a, the linear attenuation coefficients were shown after irradiation using a γ-ray source (^60^Co-γ-cell-220, Atomic Energy Commission, Canada) of various intensities (250, 500, 750, 1000, and 1500 *kGy*). The µ value decreases as the power of the gamma rays increases for all the studied GP mixes. Moreover, the blended mixes exhibited higher linear attenuation coefficients than the control mix (M0) at all the tested powers of gamma rays. According to Abdalla et al. who studied the shielding properties of granite rocks of various thicknesses against different powers of gamma rays. They found that the granite rock under study has high attenuating effectiveness and shielding properties against gamma rays at low energy^54^. To study the destructive effect of the irradiation by gamma ray, the residual strength of the tested cubes after exposure was measured. Figure 8b displayed the relation between the residual strength (RS)_rad_ after irradiation by gamma rays of intensities 500, 1000, and 1500 kGy. The control mix (M0) exhibited a declining trend in residual strength RS_rad_ as the power of the irradiated γ-rays increased. There was an 18.5–35% decrease in compression strength in the M0 mix due to the exposure to gamma rays at powers of 500 and 1500 *kGy*, respectively. This decline in strength may be attributed to the negative effect of the irradiated rays on creating a loose structure^55^. In contrast, the blended mixes containing granite powders (M20) showed an improvement in residual strength by 3% when irradiated by gamma rays of power 500 *kGy* compared to the strength measured before irradiation (the value at 28 days of curing). While irradiation by powers 1000 and 1500 kGy, the RS_rad_ decreases by 9 and 12% respectively but is still higher than those recorded by the control mix at the same powers of gamma rays. For the binary blended mixes containing LDH and granite powders, M20-LDH1 & M20-LDH2, the residual strength highly improved by 9 & 8% at 500 kGy irradiation and 2% for the power 1000 *kGy* of irradiated gamma rays. This behavior results from multiple factors that enhance the shielding properties of these blends. The primary factor is the energy storage properties of GW, which resist the destructive effects of harmful gamma rays. According to Obaid et al. and others, the granite rocks have a considerable shielding efficiency for gamma with high energy, and they can be recommended as suitable alternatives for gamma-ray shielding applications in nuclear reactors and research facilities^56^. Besides, the cross-linking occurred in the linear alumino-silicate chains in an alkali-activated matrix caused by gamma-ray irradiation^10,57^. This process leads to the formation of additional hydration products that fill the pores and enhance the compression resistance. Furthermore, the LDH nanosheets’ filling and catalytic properties, along with their ability to connect between the hydration products, enable the formation of a denser GP microstructure with higher resistance to the damaging effects of gamma rays. At the 1500 *kGy* level, these composite mixes experience a 7% and 8% reduction in compression resistance for M20-LDH1 and M20-LDH2, respectively. However, they still demonstrate a better performance than the control mix (M0, 35% loss) or their counterpart without LDH (M20,12% loss). It is worth noting that the fabricated BFS/GW reinforced by Zr–Al–CO3 LDH showed higher stability in gamma-ray irradiation than other GP composites, such as metakaolin-based geopolymer^57,58^ and slag-based geopolymer cement^59,60^.

#### Firing test

**Fig. 9:**
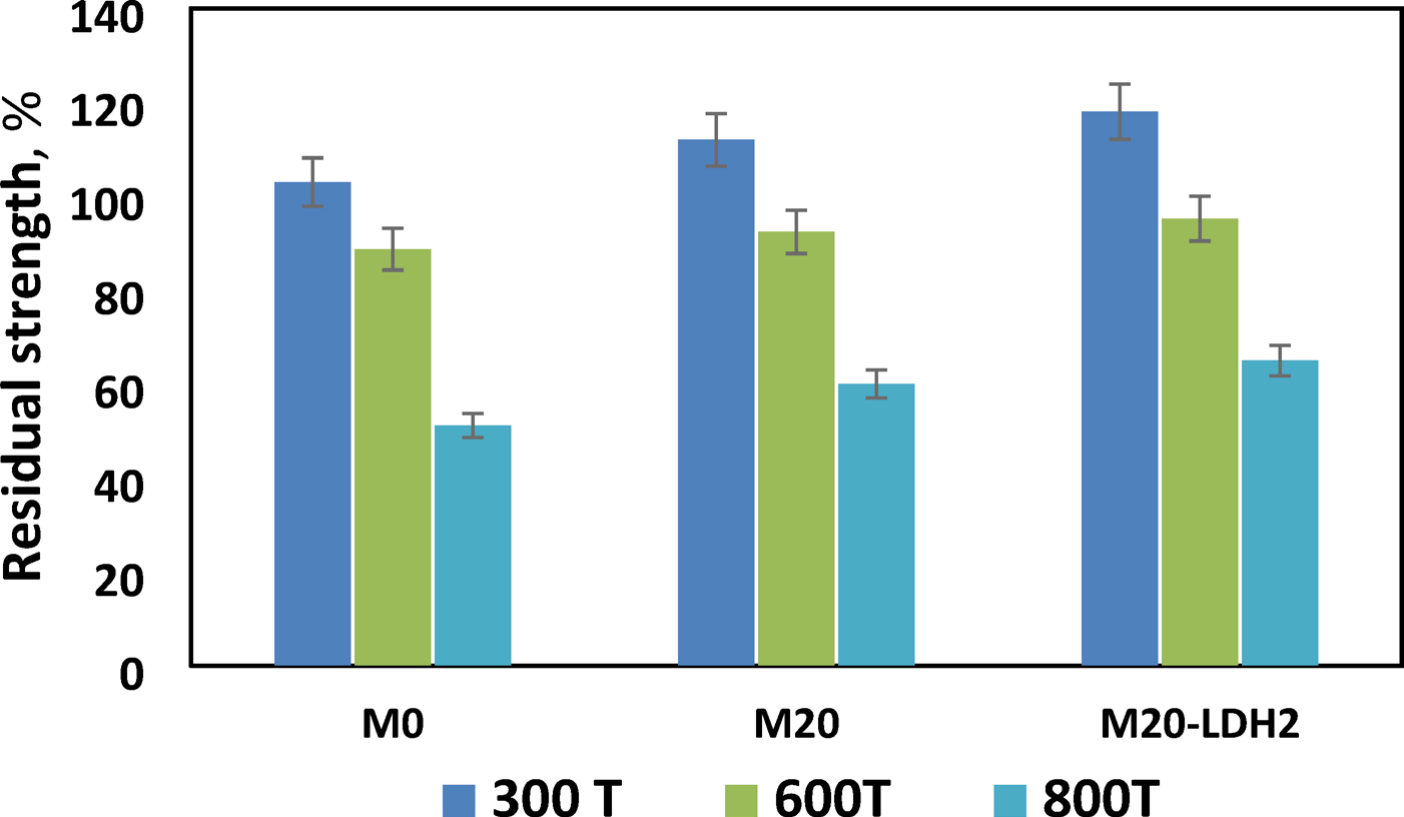
Residual strength of GP mixes after firing test.

Figure 9 illustrates the residual strength (RS%)t for specimens M0, M20, and M20-LDH2 after being subjected to firing at 300 °C, 600 °C, and 800 °C for three hours, and then cooling gradually in desiccators to room temperature. It has been observed that there is an increase in compressive strength in all the tested mixes when they are fired at 300 °C compared to their original strength (28-day values). When a hardened geopolymer mix is heated to 300 °C, the steam pressure inside the matrix increases. This steam pressure acts as self-autoclaving process that activates the unreacted geopolymer precursors leading to the progression of the alkali activation reactions and cross-linking of the geopolymer network through the alkaline hydroxyl anions^10,34,61^. These activation processes lead to the accumulation of hydrates in the pore system of the geopolymer matrix, ultimately improving its compression resistance^62^. After being fired at 600 °C, all the mixtures displayed a slightly decreased compressive strength. Among the mixtures tested, M20-LDH2 exhibited the highest remaining strength. The residual strength recorded by M20-LDH2 mix can be attributed to the combined effects of the filling and nucleation properties of LDH particles, as well as the thermal insulation properties of the fine granite powder^63,64^. The high fire resistance of the M20-LDH2 mix is still noticeable even after exposure to temperatures of 800 °C. Although the control mix (M0) showed a significant decrease in compressive strength when fired at 800 °C (RS_t_ =49.2%), the blended mixes still provided higher RS_t_ than mix M0, with mix M20-LDH2 being the best (RS = 65%). At 800◦C, dehydration/ dehydroxlation of all the formed hydrates occurred leading to the formation of micro-cracks and the opening of pores due to high pressure inside the pore matrix resulting in a great depletion in the mechanical properties and reduction in RS_t_ for all mixes. The thermal insulation properties of fine granite can reduce these destructive actions and retain some of its mechanical properties^65^. It is important to note that the firing resistance of the fabricated GP composite is comparable to that of Ordinary Portland Cement (OPC), particularly at high temperatures of 600 °C and 800 °C. According to earlier studies, OPC-hardened pastes lost 21% of their original strength at 600 °C and 47% at 800 °C respectively^58^.

## Phase composition

### XRD

**Fig. 10:**
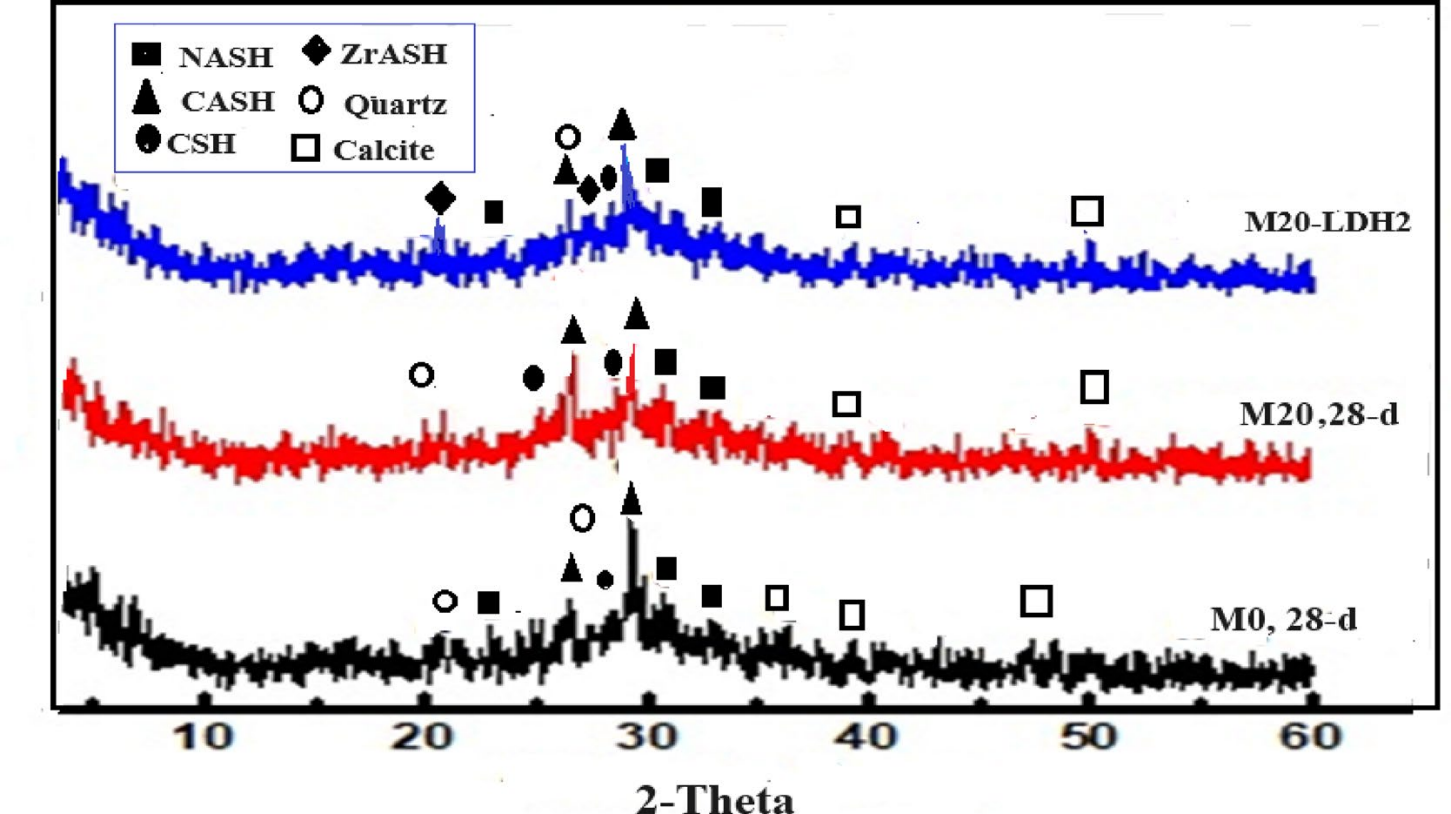
XRD of various GP formulation after 28 days of hydration.

**Fig. 11:**
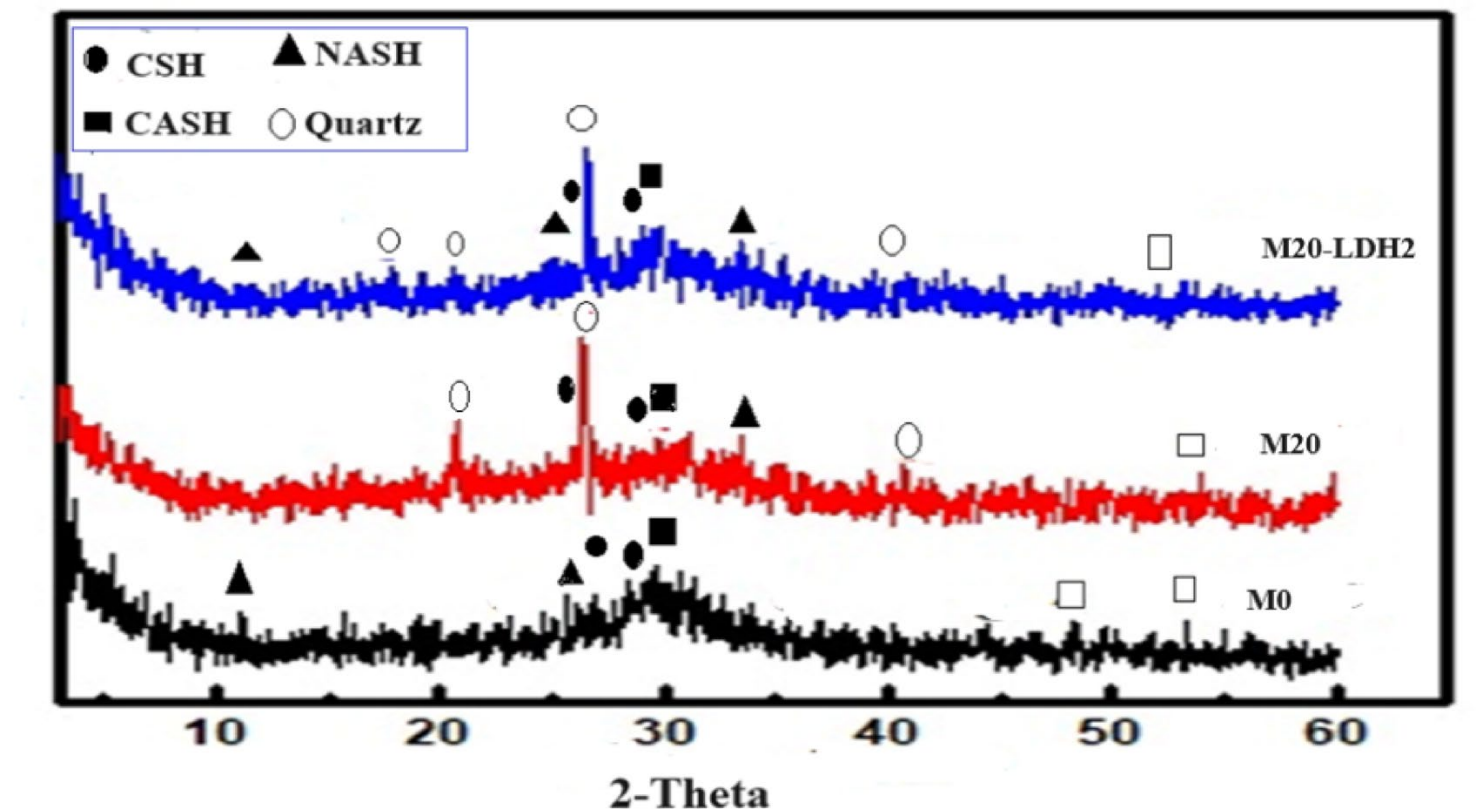
XRD of various GP formulations after firing at 800 °C.

XRD testing was conducted to identify the phases resulting from alkali-activation reactions in different GP formulations. Figures 10 and 11 displayed XRD patterns for mixes M0, M20, and M20-LDH2 after 28 days of alkali activation reactions and firing at 300 & 800 °C. According to XRD patterns, the M0 pattern showed peaks were distinguished for unreacted phases like calcite (PDF# 01–088–180) which appeared at 2θ= 36.2, 39.91, 47.1, and 50.67, quartz (PDF#01–087–2096) at 2θ= 21.4 and 26.81◦. The hydrated phases had also appeared in XRD patterns of all samples as the ill-crystalline phases of CSHs (PDF# 00–033–0306) appeared as humps at 2θ= 26.17 and 29.07° and hydrogarnet (CASHs: PDF# 00–020–0452) appeared at 2θ= 26,8 °and 31.23. Besides, a small peak appeared at 31.54 and 33.4° assigned to amorphous NASHs (PDF# 00–039–0217). These phases were generated as the main alkali activation products in the control sample in which CASH acts as nucleation sites for the geopolymerization process^10,44,66^. While, CSHs built the primary structure of solid materials, marking the start of the hardening process^67^. M20 and M20-LDH2 displayed the same hydrated phases as the control mix, M0. The main observation is the decrease in intensities of the peaks assigned to amorphous CSHs, making them less visible, while those for CASH and NASHs became more intense. The C-S-H gel was likely C-A-S-H or C-(N)-A-S-H gel, which aided in geopolymerization and enhanced the mechanical performance in the composite samples. For M20-LDH2 mix, the XRD pattern showed additional peaks assigned to Zr- alumino-silicate-hydrates (ZASH, PDF 01–088–2370) at 2θ = 22.1, 28.3° which formed due to the interaction of Zr ions liberated from the LDH with the GP matrix^64^. Zr ions can replace Ca in the CASH phase to form its analog ZrASH. Additionally, there are increased intensities of the hydrated phases in mix M20-LDH2 which confirms the effect of adding Zr–Al–CO_3_ LDH for forming more hydrated and improving the strength.

Figure 11 shows the main phases of the various mixes after firing at 800 °C for three hours. The main observation of the XRD patterns for samples fired at 800 °C is the increased intensities of the peaks assigned to unhydrous phases as; quartz and calcite and the broadening with a reduction in their intensities of the peaks of the hardened phases like; CSH, CASH, and NASH. Most of the GP hydration products were de-calcinated at this high temperature as shown in XRD patterns of all mixes. M20 and M20-LDH2 showed higher intensities for CSHs, and NASH compared to those in M0 mix, which confirms the high resistivity against firing at high temperatures for these two composites compared to the control mix. This resistance toward firing at extremely high temperatures may be related to the thermal insulation properties of granite powders (as in M20 mix) and/or the Plenty of hydration products (as in M20-LDH2 mix)^68,69^.

### Thermal study

**Fig. 12:**
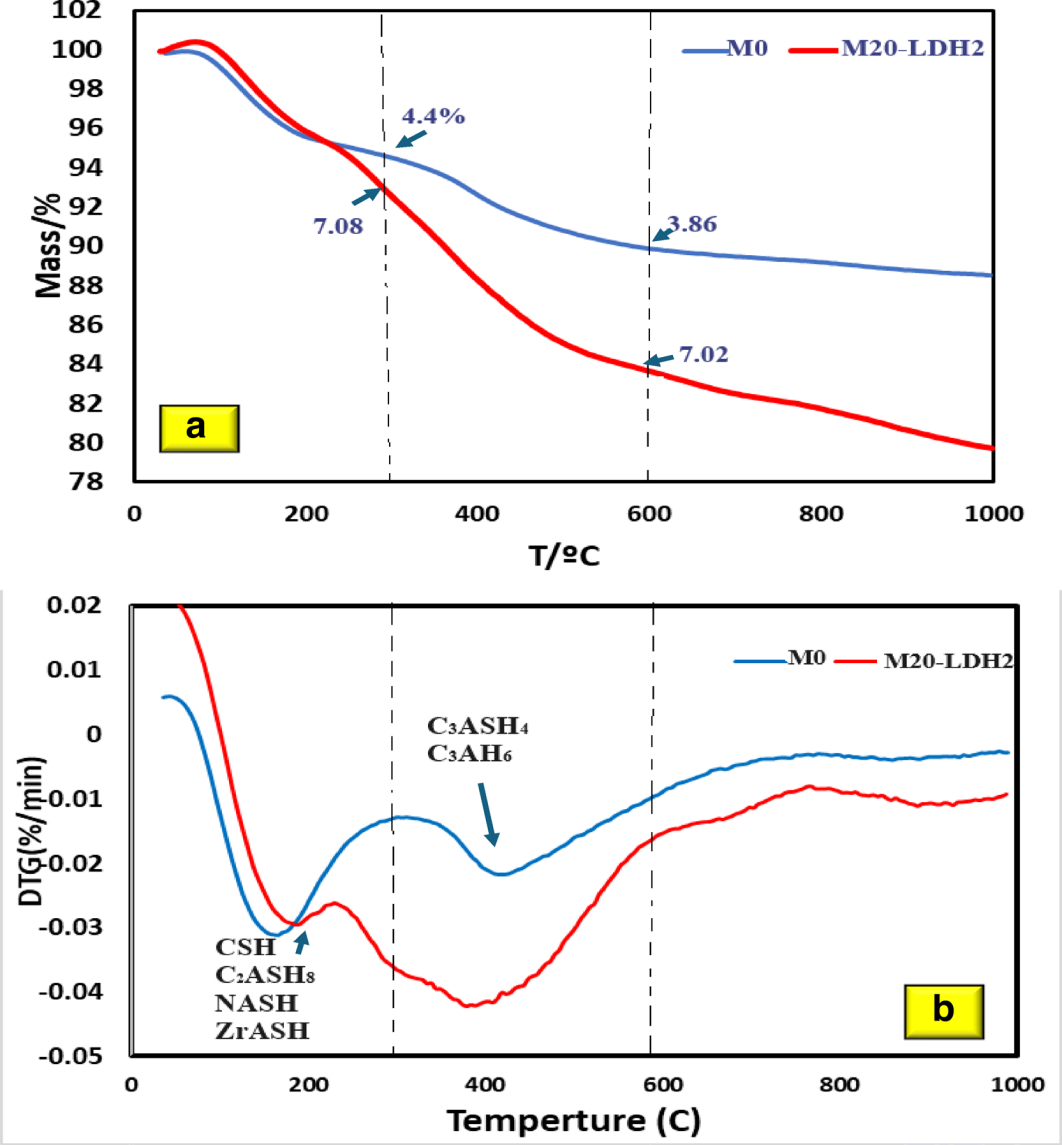
TG/DTG for M0 and M20-LDH after 28 days of curing.

Figure 12a&b shows TG/DTG curves for mixes M0 and M20-LDH2 after 28 days of curing. There is a continued decrease in mass loss (%) for the two mixes studied by heating up to 900 °C. TG/DTG curves can be divided into three stages of mass loss, at three ranges of temperatures: 50–300 °C, 300–600 °C, and 600–900 °C. The first stage of mass loss (50–300 °C) is related to the removal of water (free, physically adsorbed, and chemically combined) occurred with the dehydration of CSHs gel/semicrystalline, the disintegration of the C-A-S-H (including the gehlenite phase, C_2_ASH8), as well as the thermal breakdown of sodium -aluminate-silicate-hydrates (NASH)^70,71^. For M20-LDH, there is an increase in the percent of mass loss from 4.4 to 7.08% indicating the formation of more previously mentioned hydrates in this mix as well as ZrASH phase due to the release of Zr ions from LDH and their interaction with GP hydrates. At the second thermal decomposition stage (300–600ºC), the decomposition of the hydrocalumite, (C_4_ASH_4_) and calcium-aluminate-hydrates (C_3_AH_6_) occurred^72,73^. Again, M20-LDH2 mix recorded the highest mass loss (7.02%) while the control mix showed 3.8%. This confirms the effects of Zr-LDH in forming more hydrates which aligns with the mechanical behavior of this mix. The third stage, between 600 °C and 900 °C, is associated with the carbonation of samples during handling^74^.

The Derivative Thermal Gravimetry (DTG) study revealed two main endothermic peaks, which represent the main phases present in these mixtures. The first broad endothermic peak occurs between 50 and 220 °C, centered at 205 °C, and is associated with the continued evaporation of free water and weakly adsorbed water inside the geopolymer gel pores^75^. The second endotherm, observed at 230–450 °C with a peak intensity at 400 °C, suggests the disintegration of the most thermally stable binding phases, including C-A-S-H (hydrogarnet, C_3_ASH_4_), and the stable cubic phase of C-A-H as C_3_AH_6_^76^. M20-LDH2 showed broadening with an increased intensity in this endotherm.

### Microstructure

**Fig. 13:**
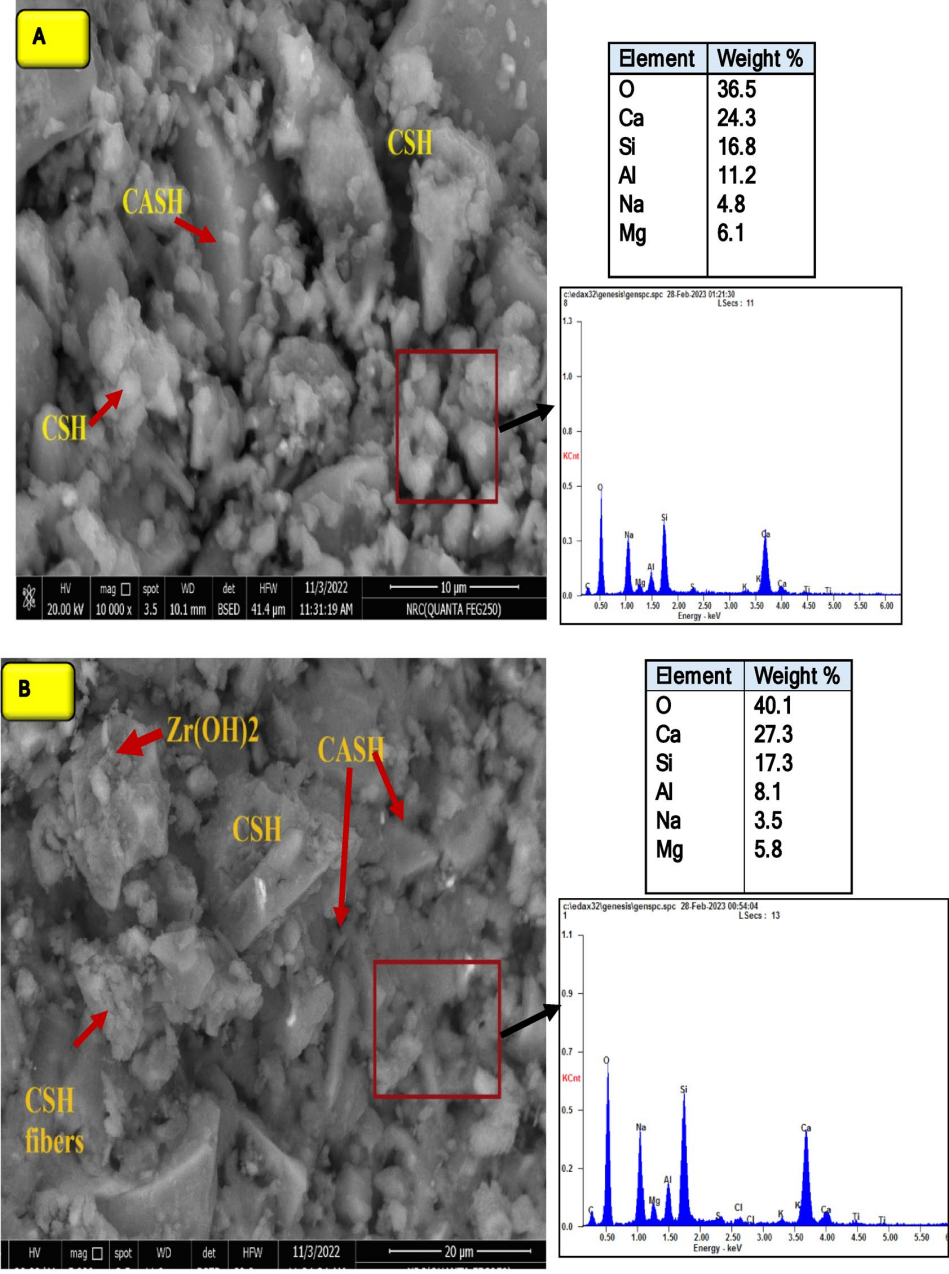
SEM/EDX analysis for (**A**) M0 and (**B**) M20-LDH2 after 28 days.

Scanning electron microscope (SEM) and energy-dispersive X-ray spectroscopy (EDX) analysis SEM were used to explore the microstructure and the morphology of the hydrates formed during the alkali-activation reaction of GP mixes. Figure 13a and b showed the SEM/EDX analysis of mixes M0 and M20-LDH2 after 28 days of curing. Notably, a compact structure composed mainly of semi-crystalline CSHs fibers which appeared as tiny rods is interconnected with pyramidal-like crystals for CASH (Al-Tobormorite) that appeared in the M0 image. These types of hydrates are responsible for the geopolymer matrix’s solidified structure, which is the main reason for the obtained strength^75,76^. EDX qualitative/semi-quantitive analysis of that image showed the chemical composition of the GP matrix composed mainly from; O, Ca, Si, Al, Na, and Mg elements with Ca/Si, Ca/Al, and Na/Al ratios: 1.45, 2.17 and 0.43 respectively. These ratios are the stoichiometric compositions of CSHs gels, CASH, and NASH respectively^77^. For mix M20-LDH2, numerous pyramidal/plate-like crystals of CASH interconnected in CSH fibers appeared in its SEM image. Besides, large quantities of stacked Zr(OH)_2_ flakes are shown, which formed due to the reaction of Zr ions with OH^−^ ions inside the GP matrix. EDX analysis of the hydrates formed in the M20-LDH2 mix showed increases in the percent of O, Ca, and Si elements with the Ca/Si and Ca/Al ratios 1.65 and 3.5 respectively. This affirms the role Zr–Al–CO_3_ LDH for catalytic alkali-activation reactions and forms extra quantities of CSH & CASH which are strength-giving phases in the GP mix.

## Conclusions

In the present research, we demonstrated the use of blast furnace slag (BFS), and granite powder waste (GW) combined with Zr–Al–CO_3_ LDH in the development of geopolymer (GP) composites with satisfactory mechanical characteristics and durable performance. The key conclusions drawn from our findings are as follows:

1. The blending of BFS with GW shortens the setting times due to the release of additional Si ions in the GP formulation which increases the water absorptivity and stiffness of the paste. Amelioration of GP mixes by Zr–Al–CO_3_ LDH greatly accelerates the setting process.
2. Up to 10% blending of GP by GW, the compression resistance improved by 7 to 10% in all the curing times, this is attributed to the presence of suitable Ca and Si ions in GP formulation for forming 2D or 3D geopolymer network for improved mechanical properties.
3. 20 and 30% replacement of BFS by GW reduced the strength due to the reduction in Ca ions via blending and formation of insufficient strength-giving phases (CSH and CASH).
4. Amelioration of GP formulation by 0.5 or 1% Zr–Al–CO_3_ LDH enhances its mechanical properties at all curing periods by about 25 to 35%.
5. BFS/GW mixes have good durability in both firing action and irradiation against gamma rays. This is related to the thermal insulation properties and the energy storage properties of granite phases.
6. The BFS/GW reinforced by Zr–Al–CO_3_ LDH showed superior resistance against the firing action and the destructive effects of irradiation by high doses (1000 and 1500 kGy) of gamma rays.
7. Such high durability is related to the combined effect of GW which acts as a thermal insulator material and energy storage material as well as the catalytic action/ filling properties of Zr–Al–CO_3_ LDH nanosheets. This combined action resulted in the formation of extra hydration products that refine the GP matrix and improve the microstructure. According to our study, geopolymer cement composites reinforced with LDH may exhibit improved properties, making them a novel and promising building material.
8. BFS/GW reinforcement by Zr–Al–CO_3_ LDH geopolymer cement can have a wide range of applications as thermal tiles, shielding applications in nuclear reactors and lining of hospital radiation rooms.

## Data Availability

The datasets used and/or analyzed during the current study are available from the corresponding author on reasonable request.
